# *Mycobacterium avium* subsp. *paratuberculosis* ELISA Responses in Milk Samples from Vaccinated and Nonvaccinated Dairy Goat Herds in The Netherlands

**DOI:** 10.3390/vetsci6020058

**Published:** 2019-06-22

**Authors:** Saskia Luttikholt, Karianne Lievaart-Peterson, Maaike Gonggrijp, Marian Aalberts, Gerdien van Schaik, Piet Vellema

**Affiliations:** 1 Department of Small Ruminant Health, GD Animal Health, P.O. Box 9, 7400 AA Deventer, The Netherlands; k.lievaart-peterson@gdanimalhealth.com (K.L.-P.); p.vellema@gdanimalhealth.com (P.V.); 2 Epidemiology Group, GD Animal Health, P.O. Box 9, 7400 AA Deventer, The Netherlands; m.gonggrijp@gdanimalhealth.com (M.G.); g.v.schaik@gdanimalhealth.com or g.vanschaik@uu.nl (G.v.S.); 3 Department of Research and Development, GD Animal Health, P.O. Box 9, 7400 AA Deventer, The Netherlands; m.aalberts@gdanimalhealth.com; 4 Department of Farm Animal Health, Faculty of Veterinary Medicine Utrecht University, Yalelaan 7, 3584 CL Utrecht, The Netherlands

**Keywords:** dairy goat, caprine, antibody response, Netherlands, ELISA, Johne’s disease, paratuberculosis

## Abstract

The aims of our study were to calculate the most appropriate cut-off value for milk samples in a serum-validated *Mycobacterium avium* subsp. *paratuberculosis* (MAP) ELISA and to analyze MAP ELISA responses in milk samples from vaccinated and nonvaccinated dairy goats in the Netherlands. Analyzed herds were representative for location and herd size of dairy goat herds in the Netherlands. A significantly higher proportion of the analyzed 49 herds were organic as compared with the total Dutch dairy goat population. First, the MAP ELISA was optimized using 992 paired serum and milk samples. At a cut-off of 25 S/P%, the relative sensitivity (Se) was 58.4% (*n* = 992, 95% CI: 48.8%−67.6%) and relative specificity (Sp) was 98.5% (*n* = 992, 95% CI: 97.5%−99.2%), as compared to serum ELISA results. The percentage of positively tested herds was 78.2% (*n* = 49, 95% CI: 63.4%−88.1%). The percentage of positive milk samples per herd (*n* = 22) was on average 4.6% (median, min, and max of 4.7%, 0.0%, and 10.7%, respectively). Average age of ELISA-positive (3.2 years) and -negative goats (3.2 years) was not different. Significantly more vaccinated goats tested positive (6.7%) as compared with nonvaccinated goats (1.1%). This study shows that a high number of vaccinated and nonvaccinated commercial dairy goat herds in the Netherlands have MAP-ELISA-positive goats.

## 1. Introduction

Paratuberculosis is a chronic enteritis of ruminants caused by *Mycobacterium avium* subsp. *paratuberculosis* (MAP) [1]. The disease is widely distributed throughout the world and causes economic losses as a consequence of long-term weight loss, reduced milk production, reduced slaughter value, increased veterinary costs, and reduced sales of breeding animals from affected herds [2,3,4].

Several diagnostic methods are currently available to detect paratuberculosis, including agent detection by PCR or fecal culture and antibody detection by, for example, ELISA or agar gel immunodiffusion test (AGIDT). Comparing the accuracy of these different tests is difficult and depends on the goal for which the results are used [5,6,7,8]. The sensitivity of immune diagnostics is generally low, mainly in early stages of infection, due to a delayed antibody response [7,9,10]. ELISA tests for MAP are commonly used because they are widely commercially available, rapid, and cheap. However, the immune response to MAP infection is paradoxical. Known from several studies in mice and ruminants, mainly cattle, there is initially a strong cell-mediated response in the subclinical stages of infection followed by strong humoral (antibody) response in late clinical stages of disease [5,11,12]. Routine assays only measure humoral response. Therefore, delayed and low antibody responses impede early detection of infected animals when using an ELISA. ELISA tests can be applied on both serum and milk samples, of which the latter are easier and cheaper to obtain [13].

Vaccination is a widely used management measure to control paratuberculosis. Meta-analyses have shown a positive effect of vaccination on delaying or reducing production losses and pathogenic effects in the majority of studies [14,15]. A vaccination campaign of 15 years in goats in Norway supports this conclusion [16]. In infected herds in the Netherlands, it is common practice to vaccinate dairy goats in the first months of life, only once with Gudair^®^ (CZ Veterinaria S.A., Pontevedra, Spain). However, limited information is available on the effect of vaccination on ELISA test results in goats, as well as on the boostering effect due to subsequent infection. Therefore, it is difficult to distinguish antibody responses due to natural infection from responses due to vaccination.

Several global prevalence studies in goats showed low animal prevalence (<16%) [17,18,19,20,21,22]. In France, the estimated true prevalence at the herd level was 62.9% and at animal level was 6.6%, based on serological testing of 11,487 dairy goats [22]. Based on 629 goat sera from 25 herds in the United States, true animal and herd prevalence rates were estimated at 1.4% (95% CI: 0.1–3.6%) and 54.7% (95% CI: 28.2–86.2%), respectively [23]. To date, prevalence studies have not been carried out in dairy goat herds in the Netherlands. True herd prevalence estimates based on serological testing in Dutch dairy cattle herds ranged from 31% to 71%, whereas animal prevalence estimates ranged from 2.7% to 6.9% [24].

The aims of our study were to calculate the most appropriate cut-off value for milk samples in a serum-validated MAP ELISA and to analyze MAP ELISA responses in milk samples from vaccinated and nonvaccinated dairy goats in the Netherlands.

## 2. Materials and Methods

### 2.1. Survey Design

The dairy goat industry in the Netherlands is increasing but was relatively small in 2013, with 400,000 goats kept in 333 herds with more than 50 goats [25]. Samples were collected from conventional and organic dairy goat farms, for which the main differences are indoor housing versus indoor housing with access to pasture, use of medication according to drug prescription law versus reserved use of medication, and feeding according to legislation versus feeding from organic sources (Stichting SKAL biocontrole (SKAL), June 2019, www.skal.nl). In 2013, 49 dairy goat herd farmers submitted 9205 milk samples to GD Animal Health, of which most samples were collected to confirm suspected or clinically affected goats. At the request of GD Animal Health, 2464 out of these, originating from 22 conventional herds, were randomly sampled. Besides 992 milk samples, 20 farmers submitted a paired serum sample from paratuberculosis-suspected goats.

### 2.2. Test

All serum and milk samples were tested using an ELISA with a relative sensitivity (Se) and relative specificity (Sp) of 64.7% and 99.2%, respectively, for bovine, ovine, and caprine serum samples as compared to fecal culture (Paratuberculosis screening ab test, IDEXX Europe B.V., Hoofddorp, the Netherlands). A specific evaluation of caprine serum samples showed a sensitivity of 82% in a group of infected goats, based on tissue culture or histopathology, and 55% in a group of negatively tested goats based on fecal culture, as well as an overall specificity of 96% [26]. The ELISA was performed according to the manufacturer’s recommendations. As the first step of the protocol, nonspecific antibodies were preabsorbed by incubating samples in a dilution buffer containing *Mycobacterium phlei* extract (milk dilution 1:2 and serum dilution 1:20). In the indirect ELISA, specific MAP antibodies were bound by an anti-ruminant IgG conjugate. Results were expressed as S/P%, which was calculated as follows: (OD value samples − OD value negative control)/(OD value positive control − OD value negative control) × 100. As no data were available for caprine milk samples, GD Animal Health calculated the most appropriate cut-off value using the abovementioned 992 paired caprine milk and serum samples. The relative sensitivity and specificity of the ELISA for caprine milk samples were calculated in comparison to the serum samples, in absence of a golden standard.

### 2.3. Data Analysis

Locations of herds at the province level in the Netherlands were available for all 333 Dutch dairy goat herds with more than 50 goats, herd sizes of 253 herds were known, and 60 out of these 333 herds were organic (Statistics Netherlands (CBS), www.cbs.nl, January, 2014). Representativeness for location and type (organic vs. conventional) of the study herds was tested using a proportion test. Representativeness for herd size of the study herds was tested with a median test. For all herds for which the percentages of positive goats were estimated, farmers were asked to provide information about vaccination (yes/no and more/less than one year ago) and the age of the goats. Differences in average age and vaccination status between positively and negatively tested goats were tested using a *t*-test and a proportion test, respectively.

Herds were classified as positive if at least 1% of the milk samples tested positive, regardless of their vaccination status. Sampling weights were considered to determine the percentage of positive herds and to adjust for the fact that the proportion of organic herds in the study population was larger than the proportion of organic herds among all dairy goat herds in the Netherlands. Sampling weights were calculated as the inverse of the herd’s sampling probability, which was calculated as the number of organic herds in the study population in relation to the number of organic herds in the sampling population. All statistical analyses were performed using STATA 13.1 software (STATA, College Station, TX, USA, 2013).

The study described in this manuscript was conducted in compliance with legislation on animal use and practicing veterinary medicine in the Netherlands. This was an epidemiological study in the field using common sampling methods for routine diagnostic purposes. According to Dutch legislation, such studies do not need approval from an animal ethics committee, but they need to be performed according to the Dutch Veterinary Practice Act. 

## 3. Results

### 3.1. Cut-Off Calculation of Milk ELISA

**Figure 1:**
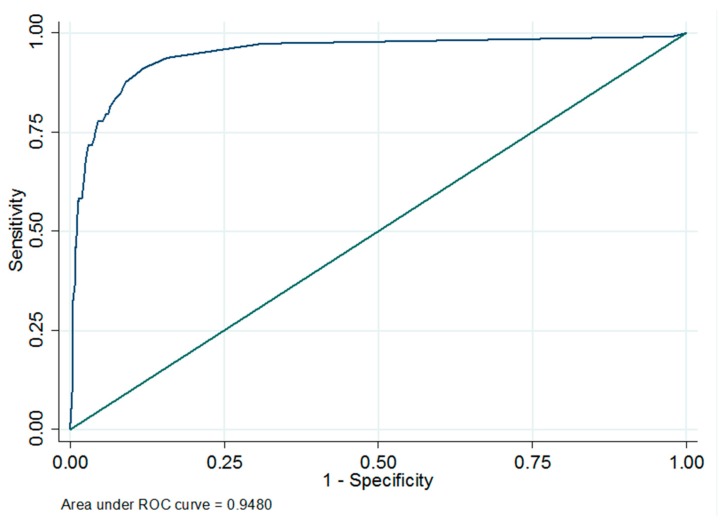
Receiver operating characteristic ROC curve and corresponding area under curve of *Mycobacterium avium* subsp. *paratuberculosis* (MAP) milk ELISA results as compared to serum ELISA results in Dutch dairy goats (*n* = 992).

For the milk MAP ELISA, a cut-off of 25 S/P% was chosen based on receiver operating characteristic ROC analysis. Using this cut-off, the Se as compared to the serum MAP ELISA was 58.4% (*n* = 992, 95% CI: 48.8–67.6%) and the Sp was 98.5% (*n* = 992, 95% CI: 97.5–99.2%) (Figure 1). The level of agreement between the level of chance of the serum MAP ELISA and milk MAP ELISA (κ-value) was 0.66.

### 3.2. Description of the Study Herds

The distribution of all participating herds per province was not significantly different from the average distribution of dairy goat herds in the Netherlands (chi-squared, *p* > 0.05). Average herd sizes (median, min, max) based on goats older than one year were 856 (707, 57, 4316) and 945 (771, 343, 4136), respectively, and these were not significantly different from a median herd size of 668 goats older than one year in the 253 Dutch dairy herds with a known herd size (median test, *p* > 0.05). The percentage of organic dairy goat herds in the analyzed 49 herds (32.7%) was significantly higher than the percentage of organic dairy goat herds in the Netherlands (18.0%: chi-squared, *p* = 0.02). For that reason, sampling weights were used to determine the percentage of positive goat herds. Only one organic dairy goat herd submitted randomly collected samples, and therefore, the percentage of positive goats per herd was only estimated for 22 conventional dairy goat herds.

### 3.3. MAP ELISA Responses on Herd Level

In total, 9205 milk samples were tested; mean number of samples (sd, min, max) per herd was 188 (352, 10, 1891). The percentage of positively tested herds corrected for the proportion of included organic herds was 78.2% (*n* = 49, 95% CI: 63.4–88.1%). There was no significant difference between the percentage of positively tested conventional and organic herds (chi-squared, *p* > 0.05). In all 27 herds in which vaccinated goats were present, at least 1% of milk samples was positive. All negatively tested herds were not vaccinated.

### 3.4. MAP ELISA Responses on Animal Level

**Figure 2:**
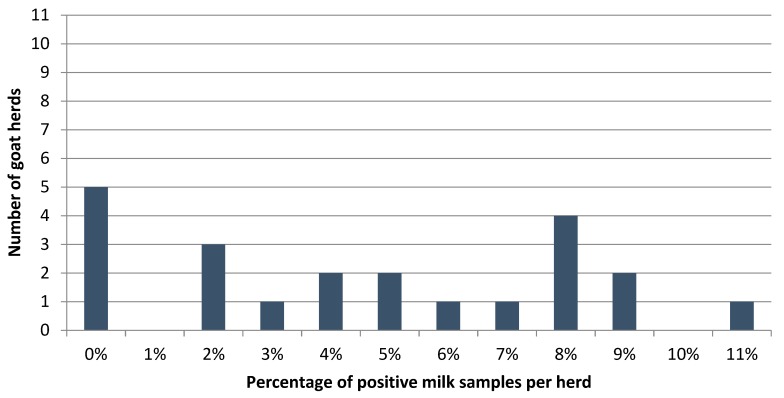
Distribution of the percentage of positive milk samples using a MAP ELISA with a cut-off of 25 S/P% based on 2464 randomly selected milk samples from Dutch dairy goats on 22 conventional herds.

The percentage of MAP ELISA positively tested goats per herd, based on 2464 randomly collected milk samples from 22 conventional herds with on average 112 samples per herd, was on average 4.6% (median 4.7%, min 0.0%, max 10.7%) (Figure 2). The percentage of positively tested herds was 77.3% (*n* = 22, 95% CI: 60.5–94.1%).

The age of all animals from 18 herds was known. Two out of these 18 herds, with only negative test results, were excluded. The average (median, min, max) ages of the positively and negatively tested goats from the remaining 16 herds were 3.2 (3.3, 1.3, 5.1) and 3.2 (3.0, 1.5, 5.0), respectively, which was not significantly different (*t*-test, *p* > 0.05).

For 2418 out of 2464 goats (93.8%), vaccination status was known; 1.1% of 900 nonvaccinated goats tested positive and 6.7% of 1518 vaccinated goats tested positive, which was significantly different (chi-squared, *p* < 0.01). Of the goats that were vaccinated less than a year before, 6.3% tested positive, and of 1470 goats that were vaccinated more than a year before, 6.7% tested positive, which was not significantly different (Table 1).

## 4. Discussion and Conclusion

Based on ROC analysis, the most appropriate cut-off value for milk samples in a serum-validated MAP ELISA was 25 S/P%, resulting in a relative sensitivity and specificity of 58.4% and 98.5%, respectively. The manual of this MAP ELISA describes as cut-offs for bovine milk: >20% to 30% is suspect and ≥30% is positive. The cut-off that we set was therefore comparable to the cut-off for bovine milk. No other studies were found in which a cut-oof has been set for the use of this kit for individual caprine milk samples.

Using this MAP ELISA in milk samples and corrected for the proportion of included organic herds, in 78.2% of the herds, at least 1% of the animals was seropositive. This can be extrapolated to the Dutch dairy goat population, as important factors such as number of goats per herd and location in the Netherlands are representative. The within-herd percentage of MAP-ELISA-positive goats was on average 4.6%, but this percentage is only representative of conventional dairy goat herds in the Netherlands.

The number of participating herds was slightly lower than needed in both sample size estimations, which resulted in wider confidence intervals. A minimum of 100 samples per herd was needed to detect with a 95% confidence level, and 21 herds did not meet these criteria. As a consequence, the percentage of positive herds could be underestimated, but as only 5 out of the 22 herds tested completely negative, the underestimation is limited. A part of the samples in this study were nonrandomly collected, which may cause selection bias [27]. Therefore, these samples were only used to estimate the percentages of positive herds.

Goat herd prevalence estimations have been studied in other countries using different diagnostic tests. These estimations may have been limited by study populations not reflecting the target populations and limited knowledge of test accuracy [28]. The following prevalence estimations were found in goat herds: Canada 83.0% (29 herds, fecal culture, ELISA, PCR) [29] and 41.4% (777 herds) [30]; Spain 52% (23 herds, AGIDT) [31]; Switzerland 23% (344 herds, PCR) [32]; and England, Wales, and Northern Ireland 1% (90 herds culture, PCR) [33]. In some other studies, prevalence rates were estimated in mixed goat and sheep herds: Portugal 27% (66 herds, ELISA) [34]; Slovenia 12% (438 herds, ELISA) [35]; Korea 18.2–38.2% (116 herds, ELISA) [17]; and France 62.9% (105 herds, ELISA) [22]. The estimated percentage of positive herds in our study was higher than in other countries. A possible explanation is that most of the Dutch dairy goats are vaccinated. These differences could also be due to the relatively large size of the Dutch herds. Furthermore, herd management in the Netherlands may be different than in other countries due to a relatively intensive housing system in which goats are mainly kept indoors.

In previous studies, different within-herd prevalence estimations were found in goats: Austria 0% (80 animals; fecal and tissue culture, ELISA) [18]; Croatia 0% (375 goats, ELISA) [20]; Portugal 1.7% (2351 sheep and goats, ELISA) [34]; Slovenia 3.5% (12,578 sheep and goats, ELISA) [35]; Korea 4.6–15.3% (582 goats, ELISA) [17]; France 6.6% (11,847 goats, ELISA) [22]; Norway 9.1% in undiagnosed herds and 3.3% in known infected herds (total of 340 goats, PCR) [21]; and Canada 35.2% (580 goats, fecal culture, PCR, ELISA) [29]. The percentage of antibody-positive goats per herd found in our study is in line with these other studies, although comparison is difficult due to the use of different tests and vaccination strategies and the use of either true or apparent prevalence.

In the Netherlands, the most frequently used MAP test is an ELISA because of the relatively low costs and high throughput. A disadvantage is the relatively low sensitivity; a low and delayed antibody response hampers early detection of infected animals [7,9,36]. Another fact explaining that animals with a measurable antibody response is low is that these measurements are performed using a diagnostic test with a serum preabsorption step during which nonspecific cross-reacting antibodies (to environmental bacteria) are removed, as they hamper diagnostic interpretation. These antibodies may, however, be important for protection, but this area is understudied and we currently do not know what constitutes vaccine-induced protective immunity other than that a correlation between vaccine-induced antibodies and protection has been observed [14,15]. Therefore, the percentage of positive herds may be underestimated.

Limited data are available on the effect of vaccination on MAP ELISA results in goats, although goat kids vaccinated at 5 days or 5 months of age had a maximum antibody response at 30 days postvaccination [37]. ELISA responses to vaccination in dairy cattle herds were detected up to 3 years or 48–60 weeks after vaccination in a Dutch and a German study, respectively [24,38]. In Australian sheep, antibody levels were detectable up to 42 months after vaccination, although boostering due to infection from the environment could not be excluded [39]. In this study, test results were not significantly different between goats vaccinated more or less than one year before, but the number of goats vaccinated less than one year ago was limited. Because the exact date of vaccination was often unknown, information on the effect of vaccination on ELISA results on a longer term is lacking. In Dutch dairy goat herds, vaccination is mainly applied after the appearance of the first clinical cases of paratuberculosis in a herd, although some farmers use vaccination as a preventative measure. Therefore, the significant difference in the percentage of antibody-positive goats between the vaccinated and nonvaccinated groups could also be explained by the fact that vaccination mostly occurs in infected herds. This assertion is further strengthened by the strong humoral response during clinical stages of disease, resulting in high antibody titers [11]. Although the vaccine does lead to sterile immunity, infection still occurs [15]. Based on recent research from Koets et al. (2019, submitted), only a limited number of goats develops an antibody response postvaccination and this response wanes during the first year. Adult goats show up to 50% seropositivity. Vaccination-induced antibodies do correlate with protective immunity (i.e., no progression to clinical disease); however, the precise mechanism of action remains to be determined.

The ages of positively and negatively tested goats were not different. Another study showed that mean S/P% in milk is higher in late lactation than at kidding; however, test performance is comparable across lactational stages [40].

In conclusion, the results of this study show a high percentage of vaccinated and nonvaccinated commercial dairy goat herds with at least one MAP-ELISA-positive goat in the Netherlands. Further research on ELISA response in relation to vaccination is recommended.

## Figures and Tables

**Table 1:** MAP ELISA results for milk samples from 1518 vaccinated and 900 nonvaccinated Dutch dairy goats, using a cut-off of 25 S/P%.

	Number	Percentage Positive	95% Confidence Interval
Vaccinated	1518	6.7%	5.5–8.1%
*More than year ago*	1470	6.7%	5.5–8.1%
*Less than year ago*	48	6.3%	1.3–17.2%
Nonvaccinated	900	1.1%	0.5–2.0%
Total	2418	4.6%	3.8–5.5%
